# Dundas et al. Respond to “Multilevel Analysis of Individual Heterogeneity”

**DOI:** 10.1093/aje/kwu109

**Published:** 2014-06-12

**Authors:** Ruth Dundas, Alastair H. Leyland, Sally Macintyre

Most research problems in epidemiology are multifaceted and, therefore, complex. The fact that they are complex should not mean that the problems cannot be researched. Statistical methodology is responding to the complexity of the research challenges.

In Merlo's invited commentary (1) on our article (2), he argues that although risk factor epidemiology can address some research questions, it is time to advance eco-epidemiology using appropriate statistical methods. Multiple membership multiple classification (MMMC) multilevel models have been around for some time (3) but are underused in social epidemiology. Multilevel models allow for individual factors (e.g., biological and lifestyle factors) and clustered groupings (e.g., neighborhoods, schools) to be studied at the same time. This enables investigation of the extent to which the different levels interact with each other or act independently of each other (4). An understanding of the factors at both individual and cluster levels and their relationships means that appropriate policy recommendations can be made (5).

Multilevel models enable separation of the contribution that each environment makes to the outcome of interest (4). There is a need to assess multiple domains of socioeconomic context (6) to enable researchers to study and identify the appropriate timing and settings of interventions to address the inequalities that exist across the life course. The social context may be relevant for the individual level but not for higher levels, or the reverse may be true. In our article, we were able to separate individual, family, neighborhood, and school factors (2) and found that family environment in childhood may be relevant for interventions to improve health in adulthood, demonstrating a key concept of social epidemiology that the clusters of individuals within families are important over and above individual effects. Clustering at the family level is frequently ignored because of the small cluster size; it can be important, as we showed, with a variance partition coefficient of 10%, despite the mechanisms remaining unknown. In addition, the consequences of ignoring family as a level on the variance partition coefficient and other general contextual effects (7) is unclear.

When interpreting the results of multilevel models, both measures of variance and measures of association are important. The use of these can provide a better understanding of the patterning of health and health inequalities (5). Measures of association such as odds ratios (specific contextual measures (7)) show how characteristics at the higher level are associated with the outcome measure. Measures of variance such as median odds ratios or variance partition coefficients (general contextual effects (7)) show the share of the variance attributable to the higher level. It is not enough to say that living in a deprived neighborhood has a negative effect on health, because living in a deprived neighborhood may be a proxy for an unmeasured individual-level factor; there is a need to identify how much of the variation is attributable to the context so that policy and interventions may be directed where they will have the greatest influence.

There is a growing need for policy to be implemented and evaluated at multiple levels of influence—individual, family, school, workplace, and community—to address both adverse health outcomes and health inequalities. Providing evidence at higher levels is potentially more helpful for public health policy than identifying individual risk factors that may have low discriminatory accuracy. Statistical techniques and models need to be available in order to do this multiple-level evaluation. We adapted designs proposed and used elsewhere in other disciplines (such as education (8) and bird ecology (9)). Our paper demonstrated the utility and adaptability of these models for epidemiology (2), and we thank Merlo for his comment that this represents a step in the right direction.
